# Functionalized Self-Assembled Monolayers: Versatile Strategies to Combat Bacterial Biofilm Formation

**DOI:** 10.3390/pharmaceutics14081613

**Published:** 2022-08-02

**Authors:** Pamela M. Lundin, Briana L. Fiser, Meghan S. Blackledge, Hannah L. Pickett, Abigail L. Copeland

**Affiliations:** 1 Department of Chemistry, High Point University, High Point, NC 27268, USA; mblackle@highpoint.edu; 2 Department of Physics, High Point University, High Point, NC 27268, USA; bfiser@highpoint.edu; 3 Department of Biology, High Point University, High Point, NC 27268, USA; hpickett@highpoint.edu (H.L.P.); acopelan@highpoint.edu (A.L.C.)

**Keywords:** biofilms, antimicrobial, bactericidal, indwelling medical devices, self-assembled monolayers, surface modification

## Abstract

Bacterial infections due to biofilms account for up to 80% of bacterial infections in humans. With the increased use of antibiotic treatments, indwelling medical devices, disinfectants, and longer hospital stays, antibiotic resistant infections are sharply increasing. Annual deaths are predicted to outpace cancer and diabetes combined by 2050. In the past two decades, both chemical and physical strategies have arisen to combat biofilm formation on surfaces. One such promising chemical strategy is the formation of a self-assembled monolayer (SAM), due to its small layer thickness, strong covalent bonds, typically facile synthesis, and versatility. With the goal of combating biofilm formation, the SAM could be used to tether an antibacterial agent such as a small-molecule antibiotic, nanoparticle, peptide, or polymer to the surface, and limit the agent’s release into its environment. This review focuses on the use of SAMs to inhibit biofilm formation, both on their own and by covalent grafting of a biocidal agent, with the potential to be used in indwelling medical devices. We conclude with our perspectives on ongoing challenges and future directions for this field.

## 1. Introduction

Bacterial infections are a growing problem in healthcare. Although antibiotics have been widely available since the 1940’s, the overuse and misuse of these life-saving drugs have led to a global increase in antibiotic resistant pathogens. In 2014, The Review on Antimicrobial Resistance estimated that annual deaths from antibiotic resistant infections would outpace those from cancer and diabetes, combined, by 2050 [1]. The global COVID-19 pandemic has likely accelerated that timeline. Worldwide, antibiotic resistant infections have risen sharply from the increased usage of disinfectants, antibiotic treatment to prevent or treat secondary infections, lengthy hospitalizations, and the prolonged use of indwelling medical devices (IMDs) such as ventilators, catheters, and IVs [2,3,4,5,6,7].

In addition to antibiotic resistance, bacterial biofilms pose a significant threat to modern healthcare. According to the National Institutes of Health, up to 80% of chronic bacterial infections in humans are caused by biofilms [8]. Biofilms are formed by bacterial cells that adhere to a surface and create a complex, three-dimensional structure encased in an extracellular matrix. The extracellular matrix is composed of many biomolecules, including carbohydrates, proteins, and nucleic acids. It helps to maintain the structure of the biofilm and likely helps resist antibiotic treatment. Biofilm-based bacteria are adept at avoiding eradication and are up to 1000 times more tolerant to antibiotic therapy than their planktonic counterparts. Bacterial biofilms are opportunistic, readily colonizing virtually any surface, especially those that are foreign to the body such as IMDs. As IMDs are increasingly utilized throughout all facets of medicine (common examples include catheters, IVs, cardiac stents, cochlear implants, orthopedic joints, and contact lenses), strategies to control biofilm formation in various IMD environments has received much attention [9,10,11,12].

Clearly, novel approaches to combat antibiotic-resistant biofilm infections are urgently needed. The development of effective inhibition strategies of bacterial biofilm formation can be a formidable challenge due to a number of factors. First, a biofilm is a dynamic entity, with bacterial cells constantly growing and dispersing, governed by complex chemical signaling pathways. Biofilm mass can fluctuate over the course of days and weeks. The inhibition of biofilm formation cannot be modeled by the simple prevention of protein or cell adhesion [13,14] because each bacterial species has its own surface characteristics that govern adhesion to a given surface.

Surface modification to prevent biofilm formation is an area of intense research, employing both physical and chemical approaches [15,16,17,18]. Surface modifications can be either preventative, by discouraging bacterial adhesion without killing the bacteria, or lethal, by promoting bacterial death. One method of chemical surface modification is the creation of a self-assembled monolayer (SAM). Self-assembled monolayers are one-molecule thick films that are attached to a surface through a covalent or similarly strong bond with a high degree of order [19,20]. In some cases, the SAM itself has been found to be effective at inhibiting the formation of a biofilm. In other cases, the SAM can be used as a covalent tether to graft an antibacterial agent such as a small-molecule antibiotic, nanoparticle, peptide, or polymer to the surface. The use of the SAM in the latter case is attractive because treated antibiofilm surfaces are residing within the body, and it is critical that any coating that is applied does not release toxic amounts of antimicrobial agents. The strong covalent bonds between the SAM and antibacterial agent prevent the release of the bactericidal agent into the surrounding medium, limiting toxicity issues for the host and the development of antibiotic resistance in the bacteria.

This review will focus on the use of SAMs to inhibit biofilm formation, both on their own and by covalent grafting of a biocidal agent such as small molecule antibiotics, antimicrobial peptides, and metal nanoparticles and cations. SAMs are also used in grafting-from and grafting-to approaches for antimicrobial polymers, but due to the vast nature and number of existing reviews on this topic, the reader is referred to the following references for a discussion of the role of SAMs in those materials [21,22,23]. 

## 2. Biofilm Formation

### 2.1. The Biofilm Lifecycle

**Figure 1:**
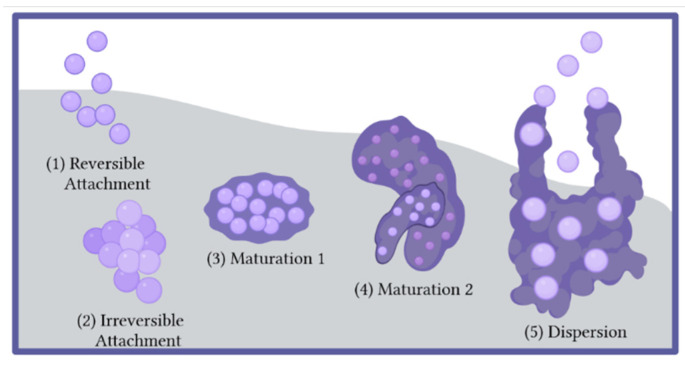
The stages of a biofilm lifecycle.

Over 99% of bacteria in the environment exist in a biofilm, yet many facets of biofilm formation and maturation are still being uncovered [24]. Bacterial biofilm formation is a complex process involving numerous systems and signaling events. Each bacterial species has evolved their own unique signals and processes, but significant crosstalk across species also exists and many medically-relevant infections may involve multispecies biofilms. In general, a biofilm lifecycle follows five discrete steps: (1) reversible attachment, (2) irreversible attachment, (3) maturation I, (4) maturation II, and (5) dispersion (Figure 1). During reversible attachment step, the bacterial cells make contact with the surface and begin to adhere, but can still be dislodged with relative ease [25,26]. Reversible attachment is generally mediated by proteins on the surface of the bacterial cell. Particular characteristics of the surface, including hydrophobicity, topography, and charge can influence the rate of bacterial adhesion, but none of these features alone has yet proven to be sufficient to prevent attachment [27]. Medical devices that are designed to promote the healing and growth of host tissues may also promote biofilm formation. Host proteins that are produced during healing, including fibronectin, vitronectin, and fibrinogen, can form a layer that is called a conditioning film. Bacterial proteins called adhesins can recognize the proteins in these conditioning films, aiding in colonization by the bacteria. Irreversible attachment follows, during which the cells associate more fully with the surface, begin to produce an extracellular matrix, and resist physical removal from the surface [25,26]. 

**Figure 2:**
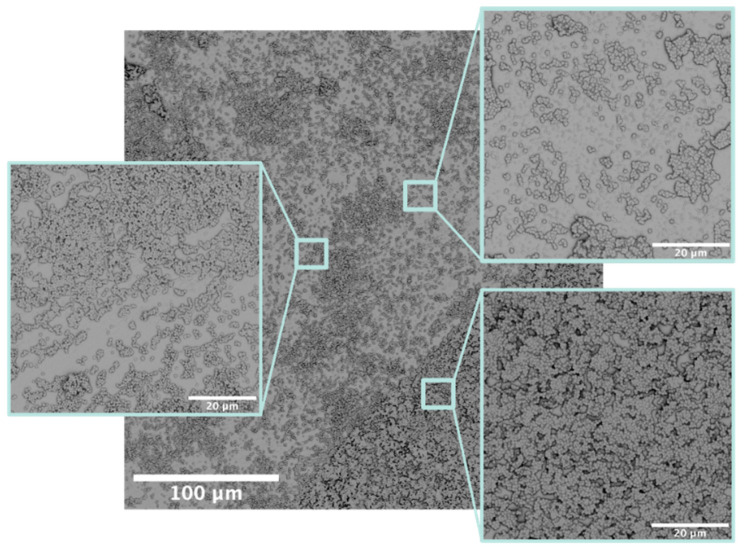
SEM images of a biofilm illustrating the different regions of two- and three-dimensional growth.

During the maturation stages, biofilms grow and become three-dimensional. Scanning electron microscopy (SEM) images of mature biofilms illustrate significant topology, including towers of various heights and water channels to allow nutrients and waste to be exchanged between the biofilms and the larger environment (Figure 2) [28,29,30,31,32]. In addition to the global changes to the biofilm structure during maturation, the bacteria in the biofilm begin to differentiate. The cells in the center of the biofilm with limited access to nutrients and oxygen often go into a dormant state [33]. These cells are known as persisters and are believed to be a major cause of the endurance and resilience of biofilm-based infections [31,34]. Bacterial persisters are metabolically dormant, but not dead [35]. If these persister cells are exposed to an environment that is more favorable, for example conditions of high nutrient availability and favorable oxygen levels, the persister cells will revert to a metabolically active state and begin to grow and divide normally. Persisters are also believed to be one of the reasons bacterial biofilms effectively evade antibiotic treatment [29,31]. As most antibiotics act on metabolically active bacteria, persisters can endure extended antibiotic treatment and return to active growth and reproduction when the therapeutic treatments are suspended.

The final stage of the biofilm lifecycle is dispersion, in which the mature biofilm erupts and disperses planktonic bacteria [29]. Biofilm dispersion is distinct from biofilm detachment, which is due to external forces, such as shear force or mechanical disruption. Dispersion is chiefly responsible for the release of planktonic bacteria into the bloodstream and colonization of distal sites with bacteria and is controlled by environmental signals and transcriptional changes within the cells themselves [36]. In large biofilms, bacterial cells experience different microenvironments depending on their proximity to the exterior or interior of the biofilm. Cells that are near the interior of the biofilm have less access to nutrients and oxygen and experience higher concentrations of waste products. It is believed that the resulting chemical gradients that are experienced by the cells across the biofilm are chiefly responsible for biofilm dispersion. Fatty acid signaling molecules, including cis-2-decenoic acid and cis-11-methyl-2-dodecenoic acid are capable of inducing dispersion of several Gram-positive and Gram-negative bacteria [37,38,39]. Hypoxic conditions and reductive stress also regularly trigger biofilm dispersion via gradients in metabolites including pyruvate, NADH, and nitric oxide [36]. While the signals that initiate biofilm dispersal are varied, one common feature is that dispersion is directly linked to reductions in the second messenger bis-(3′–5′)-cyclic-dimeric guanosine monophosphate (c-di-GMP). The levels of c-di-GMP are regulated by opposing enzyme families: the diguanylate cyclases that synthesize c-di-GMP from GTP, and phosphodiesterases that degrade c-di-GMP [40]. Enzymatic degradation of the extracellular matrix is also believed to contribute to biofilm dispersion. Significant studies of *P. aeruginosa* biofilms have identified endonucleases and glycoside hydrolases that are upregulated during dispersion and are believed to degrade DNA and polysaccharides, respectively, that make up the biofilm matrix [36]. 

### 2.2. Device Related Infections

Modern medicine increasingly relies on IMDs and surgical interventions. Both the devices themselves and the surgery provide routes for introducing foreign bacteria into the body where they can create biofilms and form a persistent reservoir for infection. Almost 80% of indwelling medical device-related biofilm infections are due to biofilms that are formed by Gram-positive *Staphylococci*, including *Staphylococcus epidermidis* and *Staphylococcus aureus* [41]. *Staphylococci* are part of the commensal bacterial skin flora, accounting for their high incidence of infection, and can be introduced through contaminated devices, from healthcare staff, or from the patients themselves [31,32]. Urinary catheters are frequently fouled by Gram-negative bacteria. *Escherichia coli* is responsible for approximately 90% of all urinary tract infections and is most likely seeded from commensal perineal flora [42]. These bacteria may be established in the bladder prior to catheterization, or they may be introduced via the catheter itself. Biofilm formation on urinary catheters, and the resulting biofilm-based bladder and urinary tract infections that they catalyze, are significant healthcare issues for patients that require long-term catheterization. Table 1 summarizes the common bacterial strains that are associated with biofilms on indwelling medical devices.

Medical devices are composed of numerous materials including plastics, metal, and ceramics. Generally, plastics are more readily colonized than metal surfaces, but bacterial biofilms can colonize both of these surfaces [42,70,71]. It is believed that the primary drivers of bacterial adhesion to medical device surfaces are van der Waals forces and hydrophobic forces [72]. The surface features including texture, hydrophobicity, and electrostatic charge all contribute to the affinity of bacteria for attachment to a surface and may even affect which strains of bacteria show affinity for a given surface.

### 2.3. Treatment Options for Biofilm-Based Infections

The diagnosis and treatment of biofilm-based infections remains difficult. Localized inflammation, pain, and symptoms of infection are often the first signs, spurring additional diagnostic testing [73]. The current standard of care for diagnosing infection of an indwelling medical device is to swab the device and culture the sample [72,74]. While this allows physicians to identify the pathogen and test for drug sensitivities, the precise nature of the biofilm contaminating the device cannot be determined without removal and extensive laboratory studies such as mechanical disruption or microscopy [74]. Due to the difficulty that is associated with definitively diagnosing biofilm-based infections, the actual incidence of these complications is likely underreported [75]. For example, infection is listed as the third leading cause of total hip replacement failure after bone lysis/loosening and dislocation according to the Australian National Joint Replacement Registry [75]. However, low-grade biofilm infections can lead to lysis and loosening of the joint, mimicking aseptic loosening that is caused by the patient’s immune response to the joint. 

There are currently no FDA-approved treatments to specifically treat biofilm-based infections. Many groups are working to synthesize and evaluate small molecules that disrupt bacterial biofilms. These compounds and strategies, while outside the scope of this manuscript, have been reviewed extensively [76,77,78,79,80,81,82]. Although these molecules remain in pre-clinical study and development, the hope is that these efforts will one day provide novel treatments for biofilm-based infections.

The current standard of care for biofilm-based infections involves extended treatment with antibiotics and, in some cases, either mechanical sanitization of the device or removal and replacement [82]. In cases such as orthopedic implants, device removal is difficult, and the replacement process is often complicated by bacterial deterioration of bone and tissue. The particular antibiotic or combination of antibiotics that are used to treat an infection depends on the bacteria that is responsible for the biofilm and its drug sensitivity profile. There are numerous reviews and reference texts that discuss different classes of antibiotics, their mechanisms of action, and their clinical uses [83,84,85,86,87,88,89]. In particular, we will highlight two classes of antibiotics that are heavily discussed below in Section 5. One common antibiotic that is routinely employed for the treatment of staphylococcal biofilms is vancomycin. Vancomycin is a glycopeptide antibiotic that disrupts cell wall biosynthesis by binding to the D-alanyl-D-alanine (D-Ala-D-Ala) terminus of peptide subunits, preventing them from being incorporated into the backbone polymers of the cell wall [83]. Vancomycin is particularly attractive as a surface-active antibiotic as it does not need to be internalized into the bacterial cell and instead sequesters D-Ala-D-Ala on the exterior of the bacteria. Antimicrobial peptides are being increasingly studied as novel therapeutics to treat bacterial infections [90]. Typically, these peptides have significant positive charges that allow them to interact with the negatively charged bacterial membrane and form pores that promote leakage of cellular contents, disruption of the cellular metabolite gradient, and cell death.

### 2.4. Characterization Methods for Biofilms

Ideally, in evaluating a surface modification for the prevention of biofilms, the treatment must be shown to be consistently applied across the surface and its impact on the bacterial biofilm lifecycle should be studied. However, the tools and methods for evaluating both surface modification and biofilm formation can vary widely from research group to research group as a function of the instrumentation that is available and the expertise of the researchers. Prior to our discussion of specific surface treatments and their impact on biofilm formation, we will quickly introduce some of the more common techniques that are employed by researchers in this area.

SEM is a common technique for the visualization of biofilm distribution, an application for which it operates on the ideal scale [91]. Specimens that are intended for viewing via SEM are often fixed with paraformaldehyde or glutaraldehyde, which cross-links the proteins, to maintain shape under vacuum conditions. Specimens should also be coated with a conductive layer (deposited via sputter coating, etc.) prior to imaging to produce high-definition images through electron backscatter and characteristic X-rays that are emitted from the specimen. This method allows for the investigation of biofilm topography and density across a surface sample. Being more qualitative than quantitative, SEM images orient the viewer to bacterial behavior in the context of clustering and biofilm spread across the sample.

Fluorescence microscopy can be used to assess antibacterial/bactericidal activity of surface treatments through colorful imaging [91]. Fluorescent microscopes use reflected ultraviolet light to illuminate the specimen across multiple wavelengths. The absorption patterns of the specimen create images that are indicative of the cell cycle state or colonization activity. LIVE/DEAD imaging uses a staining technique that fluoresces damaged and intact cells differently when fluorescence microscopy is performed. These images can be subjected to cell-counting software that determine the ratio of living to apoptotic cells across the surface sample [92]. Similarly, bacteria can be tagged with fluorochrome solutions that create visible membrane stains under fluorescence microscopy. This technique allows for an assessment of the adhesion of biofilms and visualization of bacterial distribution. 

Crystal violet staining is often implemented to quantify biofilm mass [93]. This technique allows for the stain to incorporate into sessile cells and be extracted via a variety of common laboratory solvents. Absorbance values of the solubilized wells, as measured by spectrophotometry, indicates the relative retention of biofilms across a surface. These values can be used to compare biofilm development or inhibition across multiple surface treatments. 

Colony-forming unit (CFU) counts are used to estimate the number of viable cells per milliliter [91]. The goal of this technique is to separate living from dead cells without the use of instrumentation through a series of cell stock dilutions prior to plating on growth media. This allows for plate counts to reasonably be performed by hand with enough replicates to create a reliable comparison of biofilm growth when cultured up to detectable levels.

## 3. Self-Assembled Monolayers

### 3.1. Definition and Structure of Self-Assembled Monolayers

**Figure 3:**
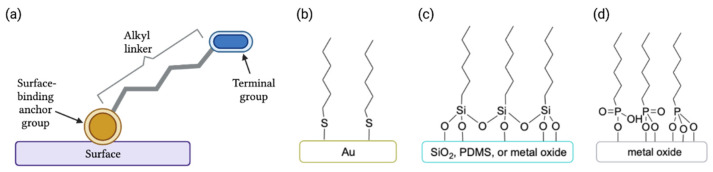
(**a**) General structure of a SAM, including the surface-binding anchor group, the linker (here an alkyl chain), and a terminal group; structure of a (**b**) thiol SAM on a gold surface, (**c**) silane SAM on a SiO_2_, PDMS, or metal oxide surface; and (**d**) phosphonic acid SAM on a metal oxide surface.

As mentioned above, a self-assembled monolayer is defined as a thin, single-layer film of small molecules that are attached to a surface in a highly ordered manner. A molecule that is capable of becoming a self-assembled monolayer is made of three parts: the anchor group, the terminal group, and the linker (Figure 3a). As they are created from a small molecule, SAMs typically are limited in thickness in the range of 1–5 nm depending on the size of the molecule, and thus fall into the category of a nanoscale material. In comparison to physiosorbed films of polymers or metals, SAMs have the advantage of resisting release into the surrounding environment due to the strong interaction of the anchor group with the surface.

The anchor group must have a high affinity for the surface, and, therefore, the class of molecule that is employed to make the SAM is determined by the surface that is used [19,20,94]. Thiols form a strong bond to gold surfaces (Figure 3b), and thiol/gold SAMs are among the most-studied SAM systems. Silanes are used with silicon, silicon oxide (e.g., glass and quartz), silicone rubbers such as polydimethylsiloxane (PDMS), and metal oxide surfaces (Figure 3c). Metal oxides also react well with phosphonic acids (Figure 3d) and carboxylic acids.

The terminal group in a SAM is selected based on the properties that are desired after functionalization. For example, if a hydrophobic surface is desired, an alkyl terminal group will be used, but if a hydrophilic surface is preferred, a polar group such as an amine or a charged group, such as an ion, will be placed at the end of the SAM chain. Alternatively, reactive functionalities may be employed as the terminal group to facilitate a reaction that achieves the desired surface chemistry. In most cases, an alkyl linker is used between the anchor and terminal groups, but other linker types (e.g., arenes, ethylene glycol oligomers, etc.) are used as well.

The stability of SAMs depends strongly on the nature of the surface-anchor group bond, the bonds in the linker and the surface-exposed terminal group, and the conditions to which the SAM is exposed. Thiols are susceptible to oxidation at the sulfur anchor group, leading to desorption from the surface, and this process is sped up by exposure to ambient air and light [95]. However, immersion in buffers mimicking physiological conditions do not appreciably accelerate oxidation and desorption of thiol SAMs, at least on short experimental timescales. A comparative study between a phosphonic acid SAM on titanium and a thiol SAM on gold in tris buffered saline (TBS) indicated that the phosphonic acid SAM quickly degraded (>80% SAM molecules desorbed within 1 day), but the thiol SAM on gold was much more quickly oxidized when it was exposed to air [96]. In air, the thiol SAM on Au suffered a complete loss of molecules in 7 days compared to a loss of only about 20% of phosphonic acid SAM molecules over 14 days. On the other hand, a silane SAM on Ti was found to be as robust as a thiol SAM on gold in TBS. Thiols show less thermal stability than silanes under vacuum conditions, probably due to the difference between a monovalent and a trivalent SAM [97]. For a detailed review of the stability of SAMs on Au, readers are referred to the following review [98]. The nature of the linker and terminal group also influences SAM stability. Longer-chain linkers have been shown to be more stable over time under physiological conditions, likely because the increased van der Waals packing between chains limits the ability of water and other chemical species to diffuse across the SAM and react with the anchor group [99,100]. Additionally, under ambient conditions, terminal amine groups can be oxidized to amides, thus changing the nature of the surface chemistry [101], and similar processes likely occur in other functionalized SAMs. Finally, one limitation of SAMs is their low durability in tests of mechanical robustness [102,103], which is an important consideration for their implementation in high-stress IMDs such as orthopedic implants.

### 3.2. Surface Characterization Methods for SAMs

The thin film thickness of a typical SAM can make analysis of surface coverage challenging. Sensitive techniques that can be applied to a surface are needed. The specific techniques vary as a function of the expertise of the researchers and the instrumentation that is available.

X-ray photoelectron spectroscopy (XPS) is a powerful technique that can be used to quantitatively determine the elemental composition of the surface [104]. X-rays are used to irradiate the surface and the binding energies of the ejected electrons are quantitatively measured. The resulting peaks are characteristic not just of the elements that are present, but also of their chemical environment and oxidation state. Therefore, this technique is especially useful when the SAM is formed and then further reacted, as it can detect those subtle changes. Drawbacks to the technique include the destructive nature of the measurement to the sample, and the fact that this sophisticated instrument requires expertise to operate correctly.

Surface-sensitive modes of infrared (IR) spectroscopy is another instrumental technique that is commonly employed to probe for certain types of bonds [105]. As most surfaces that are used for biofilm evaluation are strong absorbers, IR methods using reflectance such as attenuated total reflectance (ATR) and infrared reflection-absorption spectroscopy (IRRAS), also known as ranging angle infrared reflectance spectroscopy (RAIRS), are the most appropriate. ATR is available as an attachment for most laboratory-grade IR spectrometers, and its use with a wide variety of samples and the subsequent data analysis is straightforward.

Measuring the water contact angle (WCA) of a functionalized surface is one of the simplest techniques that can be used to assess the success of surface functionalization as a function of changes of hydrophobicity and hydrophilicity and is frequently used as a proxy for surface energy in different situations. The relationship between WCA and the adhesion of various bacteria to a surface has been extensively studied [106]. This technique is not very precise with regards to the location on a substrate, but it is quick and can be done with a homebuilt apparatus [107], making it very accessible to most laboratories.

Atomic force microscopy (AFM) and SEM are microscopy techniques that are sometimes employed to characterize surface morphology as a result of SAM functionalization. AFM is a very sensitive technique that can detect changes in the heights of only a few nanometers, making it capable of detecting a SAM, especially if a contrasting pattern is present [104]. The scale of a typical SEM ranges from hundreds of nanometers to microns, and so it is unable to detect the monolayer on its own. However, it is sometimes used to characterize larger-scale morphological changes in the surface.

## 4. Preventative SAM Strategies for Biofilm Inhibition

A preventative strategy relies on the change in the physical surface properties as a result of SAM modification to inhibit bacterial adhesion, disrupt biofilm formation, or promote biofilm removal to prevent the build-up of a biofilm layer [108]. In contrast to lethal strategies that actively kill bacteria, discussed in Section 5, a preventative strategy is more passive. Table 2 summarizes the structures that are used as preventative SAMs.

### 4.1. Prevention of Bacterial Adhesion by Increasing Hydration of the Surface

An early study in 2007 conducted a head-to-head comparison of a hydrophobic alkyl SAM to a more hydrophilic NH_2_-terminated alkylsilane SAM on silicon wafers to determine the influence of surface wettability on biofilm formation (Table 2, row 1) [108]. Interestingly, while both surfaces had similar amounts of *Escherichia coli* bacterial cells adhered, the morphologies of the resulting biofilms were different as determined by fluorescence microscopy and SEM. The amine-terminated SAM resulted in more branched networks of cells while the alkyl SAM caused the cells to cluster. Furthermore, the wettability of the surface also influenced the time intervals of biofilm development over the two-week period they were observed.

That same year, it was found a tri(ethylene glycol) (TEG)-terminated SAM on Au was more effective than a carbohydrate L-gulonamide-terminated SAM at inhibiting biofilm formation of fluorescently labelled *E. coli* using confocal scanning laser microscopy (CSLM) (Table 2, row 2) [109]. This finding is consistent with earlier studies that showed that ethylene glycol oligomers prevented bacterial cell adhesion but not biofilm formation [112,113]. As both the TEG- and L-gulonamide-terminated SAMs have similar water contact angle (WCA) measurements, this difference in activity highlights that biofilm inhibition cannot simply be attributed to differences in hydrophilicity/phobicity of the surface. Further investigation by administering the TEG molecule in vitro to the bacteria showed that TEG impacted *E. coli* motility, thus concluding that its superior performance on the surface may be attributed to both prevention of bacterial adhesion and control of bacterial motion in solution. However, in a subsequent study, a different carbohydrate-terminated SAM that was based on D-mannitol was found to be more effective than the TEG-terminated SAM at inhibiting biofilm development of *E. coli*, *Pseudomonas aeruginosa*, and the fungus *Candida albicans* [114]. Whereas the TEG-terminated SAMs inhibited biofilm growth up to 7 days, inhibition by the D-mannitol-terminated SAM lasted for the experimental period of 26 days. This success with D-mannitol was followed up by exploring the impact of polyol chirality on biofilm formation [115]. It was found that *E. coli* cells initially adhered to the polyol SAMs, but once biofilm formation began, they selectively grew in the alkanethiol regions rather than in the polyol background. Interestingly, racemic mixtures of the chiral polyol SAMs were observed to be more effective at inhibiting biofilm growth than either enantiomer alone, which hints that closer packing in the water solvation layer may be driving the biofilm inhibition.

In the studies that were discussed above, the authors employed a technique that is known as microcontact printing [116] to pattern regions of non-biofilm-resistant alkyl SAMs against a background of their bioinert SAM. These studies corroborated the efficacy of TEG-, D-mannitol- and polyol-terminated SAMs, respectively, even resisting spillover of 60 μm-thick biofilm from the adjacent non-bioinert region [114]. This group has also demonstrated the interesting patterning capabilities of SAMs by creating gradient surfaces in which the concentration of the bioinert TEG-terminated SAM decreases controllably across the surface as the concentration of an alkyl SAM increases [117]. It was shown that the ability of the surface to resist biofilm formation decreased linearly as the concentration of the TEG-terminated SAM decreased. This behavior is in contrast to the adhesive behavior of mammalian cells, which need a certain critical threshold of TEG concentration on the surface to inhibit cell adhesion. This data points to a non-specific adhesion mechanism for *E. coli* biofilms on a surface. 

Ulvans are polysaccharides that can be isolated from seaweed, and, therefore, contain many hydrophilic hydroxyl groups. There are two ulvans that were isolated from two seaweed species that were grafted onto titanium using amide coupling to an amine-terminated SAM [118]. The ulvans seemed to prevent bacterial spreading of *P. aeruginosa* across the surface but biofilms with significant thickness were still achieved. As *P. aeruginosa* is a motile bacteria species, the impact of the ulvans on non-motile *S. epidermidis* was also investigated, and a pronounced anti-adhesive effect was observed. In a follow-up article, an excess coupling reagent was used to cap all the carboxylate groups of the ulvans so that the resulting charge was influenced to a larger extent by sulfate groups prior to grafting to silicon wafers [119]. The highest molecular weight ulvan with the higher proportion of sulfate groups was the most effective at preventing *S. aureus* colonization. The authors found no evidence of bactericidal activity and attributed the effect of the ulvan-functionalized surfaces to preventing the bacteria from adhering.

Zwitterionic organic materials have been highly investigated as anti-fouling materials. When grafted to a surface, zwitterions can create a dense hydration layer that has been shown to suppress protein and bacterial adhesion [14]. Zwitterionic SAMs were compared to zwitterionic polymers by their ability to inhibit *S. epidermidis* and *P. aeruginosa* biofilm formation on Au surfaces under flow conditions (Table 2, row 3) [110]. The study looked at an oligo(ethylene glycol)-terminated SAM and a mixed SAM that was composed of two alkanethiols, one bearing a negatively-charged sulfonate headgroup and the other a positively-charged tetraalkylammonium headgroup. While these SAMs performed comparably to the zwitterionic polymers in inhibiting *S. epidermidis* adhesion in a short 3-h study, the SAMs fell short of the polymers’ performance in biofilm formation over a 48-h period. For *P. aeruginosa*, the SAMs were less effective than the polymers in both the short adhesion study and the longer biofilm formation study, although the mixed zwitterionic SAM qualitatively performed better than the OEG-terminated SAM. The authors speculated that the lower performance of the SAMs compared to the polymers was likely due to a combination of a thinner film thickness and oxidation of the sulfur anchor leading to SAM loss at the surface, although they did not account for other factors such as the density of charged groups that vary between the systems.

In a separate study, PDMS was functionalized via hydrosilylation with allyl carboxybetaine to present a zwitterionic group at the surface (Table 2, row 4) [111]. A comparison of the carboxybetaine-functionalized PDMS to native PDMS showed that the surface functionalization significantly decreased the colonization of *E. coli* and *S. aureus* over a 24-h period. This is true even if the functionalized PDMS had been aged for 3 months. It was speculated that the hydration of the zwitterionic-capped surface suppressed the ability of the bacteria to adhere to the surface.

### 4.2. Prevention of Biofilm Formation by Interruption of Quorum Sensing

**Figure 4:**
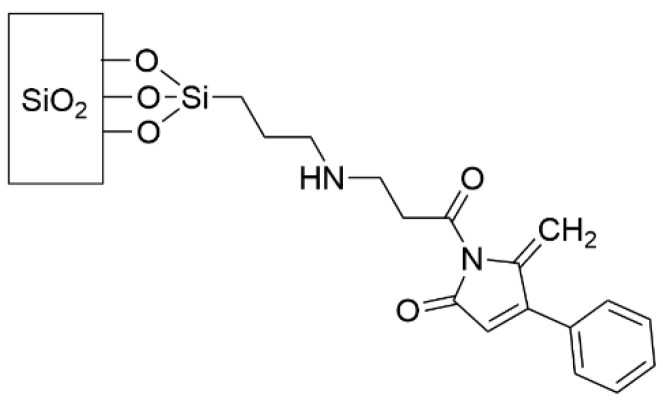
Example of DHP-containing SAM that is effective against *P. aeruginosa* and *S. aureus* in reference [120].

Quorum sensing (QS) is a chemical communication method that is used by bacteria to sense and communicate environmental conditions and coordinate a group response. In the context of biofilm formation and maintenance, QS is critically important for such activities as secreting the extracellular polymeric substances (EPS) to form biofilms and to begin excretion of virulence factors [31,76]. Therefore, grafting a molecule that disrupts QS pathways holds potential to suppress biofilm formation. A variety of dihydropyrrole-2-ones (DHPs), which are known to interfere in the quorum sensing system that is regulated by N-acyl homoserine lactones (AHLs) in Gram-negative bacteria, were grafted to a glass surface via covalent attachment to a SAM of (3-aminopropyl)triethoxysilane (APTES) and shown to suppress the number of *P. aeruginosa* and *S. aureus* cells that were adhered to the surface by up to 97% after 24 h (see Figure 4 for an example of an effective DHP-containing SAM) [120,121,122]. Structurally-related furanones were also found to be effective at suppressing *P. aeruginosa* and *S. aureus* biofilm formation [123]. The proportion of dead bacterial cells was unaffected by the surface treatment, suggesting that the mechanism of biofilm inhibition is either by causing the bacteria to remain in the planktonic phase or by promoting more cell detachment from the surface rather than by killing the cells. Additionally, it is interesting that the Gram-positive *S. aureus* are similarly affected by the presence of these molecules on the surface, despite using different QS molecules for their intraspecies communication.

The inhibition of the quorum sensing pathway was also the goal for grafting the *P. aeruginosa*-derived Ntn-hydrolase PvdQ on PDMS [124]. PvdQ cleaves the ester bonds in AHLs, thereby linearizing the molecules and rendering them inert. The authors hypothesized that this approach would functionally remove AHLs in situ and thwart the QS and coordination that is necessary to form mature, adherent biofilms. PvdQ was coordinated to an APTES-functionalized PDMS surface, which inhibited biofilm formation of *P. aeruginosa* by 50% as compared to the unfunctionalized PDMS control. This result suggests that this approach could be a viable strategy for limiting biofilm formation on surfaces.

## 5. Bactericidal SAM Strategies

In addition to inhibiting bacterial biofilm formation themselves, the terminal functionality of a SAM can be used to hold a bactericidal entity on the surface. Most bactericidal SAMs function via a killing-by-contact mechanism, utilizing bactericidal agents that operate at the bacterial exterior. For these surfaces, the bacteria are killed when they contact the treated surface. The covalent tether between the surface and the bactericidal agent is particularly attractive due to the ability to prevent release that can lead to the development of bacterial resistance and the ability to employ relatively low concentrations of the active material compared to administration in vivo. For bactericidal agents that require internalization into the bacterial cell to operate, these can be used in a SAM, but the mechanism of action is killing-by-release, meaning that the SAM releases the bactericide over time. Drawbacks of this strategy are the potential issues with concentration control of the bactericidal agent, leading to toxic effects if too much is released early in its use and a lack of activity once the coating has lost its bactericidal agent. Both of these strategies will be discussed in the subsections below.

### 5.1. Quaternary Ammonium SAMs

3-(Trimethoxysilyl)-propyldimethyl-octadecyl ammonium chloride (Si-QAC) was among the early SAMs that were studied for biofilm inhibition (Table 3, row 1). In 2002, silicone rubber discs that were coated with this SAM and seeded with *S. aureus* were subcutaneously implanted in rats and evaluated for biofilm formation at 3 and 7 days versus uncoated control discs [125]. The SAM-coated discs were found to have only a few viable bacteria after 3 and 7 days in comparison to the control discus. In a separate study, the same SAM was applied to titanium, and its inhibitory effect towards *Streptococcus mutans*, the pathogen that causes dental cavities, was investigated [126]. Bacterial colonization was found to be suppressed, although not as effectively as it was for the fungus *C. albicans*. The Si-QAC titanium was found to inhibit colonization by killing bacteria rather than simply by changing the hydrophobicity to alter cell adherence. Further, it was found that the long alkyl chain on the quaternary ammonium species was important for suppressing bacterial adherence to the surface.

Quaternary ammonium-containing thiols with different structures were evaluated for microbicidal activity of *S. aureus* on a gold surface (Table 3, row 2) [127]. There were two different linker types that were used and one of the quaternary ammonium chains on the head group was varied in length. The use of a linker with a secondary amide that is capable of hydrogen bonding coupled with a long (C_10_ to C_14_) alkyl chain on the ammonium group had the highest degree of antimicrobial activity but lost this activity after 24 h due to the accumulation of dead bacterial cells on the surface. 

Titanium discs were functionalized with APTES and *N*,*N*-dimethylaminopropyltrimethoxysilane, and then derivatized to terminate in a variety of quaternary ammonium salts (Table 3, row 3) [128]. These SAMs were incubated with Gram-negative *E. coli* and Gram-positive *S. aureus* for 24 h, and the amount of adhered bacteria was evaluated using CFU counts. For both species of bacteria, the **Ti-AUTEAB** outperformed **Ti-OA** and the other SAM types in preventing the adhesion of viable bacteria to the surface.

### 5.2. Small Molecule Antibiotic- and Antiseptic-Terminated SAMs

Clinically, small molecule antibiotics are the first line of defense for managing bacterial infections. Unfortunately, bacteria have evolved savvy mechanisms of resistance to many of these drugs over their decades of use, which are often tied to the practice of antibiotic overuse in high concentrations. Covalent tethering of these molecules to a surface has promise in combatting biofilm formation because it employs much lower overall concentrations of antibiotic while keeping the antibiotic at the position of the biomedical implant. 

**Figure 5:**
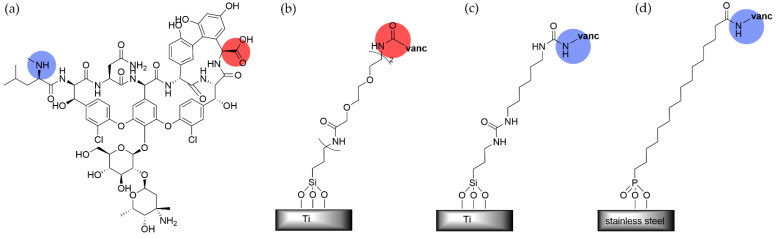
(**a**) Structure of glycopeptide antibiotic vancomycin with the grafting points highlighted with a blue circle for the secondary amine and a red circle for the carboxylic acid. (**b**) Covalent tethering of vancomycin to titanium through the carboxylic acid in references [129,130,131] and (**c**) to titanium through the secondary amine in reference [135] and (**d**) to stainless steel through the secondary amine in reference [136].

Vancomycin is a powerful glycopeptide antibiotic for combatting Gram-positive bacteria such as methicillin-resistant *S. aureus* (MRSA). Conjugation of this drug to a surface is, therefore, an attractive strategy for combatting biofilm formation. As vancomycin contains both a free carboxylic acid and a secondary amine (Figure 5a, red and blue circles, respectively), different tethering strategies have been used by different groups.

Several papers have been published on the covalent attachment of vancomycin through its carboxylic acid to the surface of titanium alloys via a silane-based SAM (Figure 5b), and it was found to be effective at inhibiting Gram-positive bacteria *S. aureus* and *S. epidermidis* from forming colonies and subsequent biofilms [129,130,131]. This effect was maintained after multiple challenges of the same substrate with *S. epidermidis*, and after exposure to serum proteins. The vancomycin-tethered monolayer was found to maintain its activity after multiple press-fit insertions into a cadaveric rat femora, indicating its mechanical integrity [129]. Interestingly, *S. aureus* did not exhibit signs of vancomycin-resistance when exposed to surface-grafted vancomycin. When a vancomycin-coated titanium was evaluated in conjunction with in vitro rifampicin against *S. aureus*, the bacteria also did not develop rifampicin resistance and decreased the rifampicin minimum inhibitory concentration [132]. As expected, however, this material was not as effective against Gram-negative *E. coli*. Vancomycin-tethered titanium has been used in rat [133] and sheep [134] animal models to reduce the signs of clinical infections, and in the case of the sheep, promoted bone-healing despite being challenged with *S. aureus*. This indicates that an 80% inhibition of biofilm formation is sufficient to translate to better clinical outcomes. 

An alternative method of tethering vancomycin to titanium through a silane SAM was developed that could be generated without an inert atmosphere, and thus was more applicable to scale-up of the implant coating process [135]. The grafting point was through the secondary amine of vancomycin (Figure 5c). This vancomycin-linked SAM was applied to a mouse model and displayed a high level of *S. aureus* biofilm inhibition as in the previous studies.

Vancomycin was also grafted through its secondary amine to stainless steel 316L via a phosphonic acid headgroup (Figure 5d), and its performance was compared to simple alkyl and OEG SAMs and to a tethered version of aminoglycoside antibiotic gentamicin [136]. Not surprisingly, the vancomycin and gentamicin SAMs outperformed the preventative alkyl and OEG SAMs, the latter of which did not statistically differ from the unfunctionalized stainless steel control. After 24 h, the gentamicin-linked SAM lost its effectiveness while the vancomycin-linked SAM continued to inhibit biofilm formation for up to 48 h. The authors speculate that the lack of action of the SAMs on stainless steel as compared to similar SAMs on gold or silicon surfaces may be due to stainless steel’s increased surface roughness. However, when the phosphonic acid SAM was linked to the powerful antibiotics, gentamicin and vancomycin, the biofilm thickness was reduced by 70% and over 99%, respectively.

Daptomycin is a lipopeptide antibiotic that, similar to vancomycin, is active against Gram-positive bacteria. A cysteine-functionalized daptomycin derivative was grafted to a titanium alloy surface through a silane SAM via a multi-step process [137]. *S. aureus* colony formation was inhibited by 72% and 54% at 8 h and 24 h, respectively as compared to the control. 

Tetracycline is a broad-spectrum antibiotic that is active against both Gram-positive and Gram-negative bacteria, unlike vancomycin and daptomycin. A multistep process was used to graft it to a Ti surface using a silane SAM [138]. Incubation of the tetracycline-functionalized rods with *E. coli* showed no bacterial colonization after 24 h by fluorescence microscopy, and further bacterial challenges showed that the surfaces also partially inhibited *S. aureus* colonization.

The antiseptic chlorhexidine was grafted onto an aminosilanized titanium surface using glutaraldehyde as a linker to condense with both the amine headgroup of the SAM and guanidine moieties of the chlorhexidine [139]. The resulting surfaces inhibited *S. aureus* biofilm formation proportionally to the concentration of chlorhexidine that was used to treat the surface. The coating was found to be more effective than the control at periods of 3 and 7 days, but the level of antimicrobial activity did decrease over time, perhaps due to hydrolysis of the imine groups that were used in the antiseptic tethering.

Taken together, tethering small-molecule antibiotics and antiseptics to a surface has shown great promise, including high efficacy in animal models. Furthermore, this strategy seems to suppress the development of antibiotic resistance in situations where global administration of the antibiotic drug would lead to resistant strains.

### 5.3. SAMs Promoting Release of Anti-Microbial Agents

**Figure 6:**
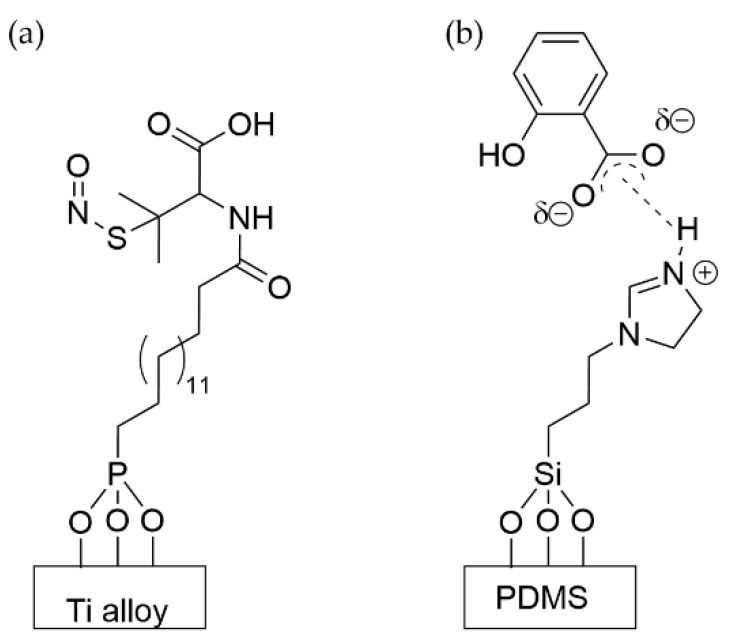
Structures of (**a**) NO-releasing [140] and (**b**) salicylic acid-releasing [141] SAMs.

Broad spectrum small molecule antibiotics have also been grafted to SAMs with groups that are labile under the physiological conditions in which a biofilm may grow. Cleavage of the tether results in the release of the antibiotic for bactericidal action. S-nitroso-penicillamine was coupled to a 16-phosphonohexadecanoic acid SAM on a titanium alloy to function as a nitric oxide (NO) donor (Figure 6a) [140]. When *E. coli* and *S. epidermidis* were exposed to this SAM, they showed a mild reduction in growth. A combination of the SAM exposure and in vitro administration of tetracycline was able to reduce the growth of *E. coli* as compared to tetracycline alone, but not of *S. epidermidis*. 

Salicylic acid, a broad-spectrum biocide, was coordinated electrostatically to amine-terminated silane SAMs on PDMS that was made from APTES and 3-(2-imidazolin-1-yl)propyl triethoxysilane (IPTES) (Figure 6b) [141]. The salicylic acid was released into the medium and showed an inhibitory effect against the viability and growth of sessile *E. coli*, *S. aureus*, and *S. epidermidis* of up to 90%. While these functionalized surfaces will retain their antibiotic activity for a limited time, this may be appropriate for the implementation of a surface in a situation with a limited exposure window, such as healing post-surgery.

### 5.4. Anti-Microbial Peptides Grafted to SAMs

Many organisms have evolved antimicrobial peptides (AMPs) to combat infections by bacteria, fungi, viruses, and other microbes [142]. While the structural diversity of these molecules is immense, in general AMPs are short (10–25 amino acids) and have cationic groups that are attracted to the negatively-charged outer membranes of the microbes, ultimately leading to membrane disruption and lysis. Derivatives of naturally occurring AMPs as well as fully synthetic AMPs have been covalently tethered to a variety of surfaces through SAMs [143,144]. One important point to consider about grafting antimicrobial peptides is that by tethering the peptide to the surface, the mechanism of action is likely impacted. For example, in vitro gramicidin A acts by forming dimers that create transmembrane channels that lead to cell lysis and death [145]. However, if the peptide is grafted to the surface, dimer formation is likely not possible and thus the antimicrobial mode of action is likely different. Therefore, for many of the references that are discussed below, the determination of the mechanism of action is ongoing.

One of the first AMPs to be covalently grafted to a surface through a SAM was magainin I, using a carboxylic acid-terminated alkanethiol SAM on Au (Table 4, row 1) [146]. It was evaluated for biofilm inhibition of *S. aureus*, *Enterococcus faecalis*, and *Listeria ivanovii*. The results indicate that the presence of the covalently immobilized magainin peptide reduces bacterial cell adhesion by 50% in comparison to the control carboxylic acid-terminated SAM. Using a LIVE/DEAD stain furthermore indicated a higher proportion of dead bacteria on the magainin-functionalized surface than the control. However, in enumeration experiments on the adhered bacteria, they continued to grow once they were removed from the magainin I-functionalized surface. Therefore, the authors postulate that the mechanism of magainin I is actually bacteriostatic, meaning that the magainin I surface does create enough pores in the bacterial membrane to disrupt growth, but not so many as to completely lyse and kill the cells. 

Magainin I has five different amines that can be used to conjugate to the carboxylic acid SAM, thus leading to different presentations of the peptide on the gold surface. Thus, the researchers followed up the study above with a similar one using the AMP gramicidin A, which can be selectively grafted to a surface through reductive amination of its formylated N-terminus with an amino-terminated alkanethiol SAM (Table 4, row 2) [145]. This modified surface was able to inhibit biofilm formation up to 24 h, and qualitatively its antimicrobial activity was higher than magainin I. Similar to magainin I, the authors postulate the mechanism of action is bacteriostatic rather than bactericidal.

Another antimicrobial peptide, temporin Sha, and its derivatives have also been investigated for antimicrobial activity. Exposure of *L. ivanovii* to a temporin Sha-functionalized gold surface led to significant morphological changes of the individual bacterial cells and a killing efficiency of over 75% compared to less than 10% for the control in biofilm formation after 3 h (Table 4, row 1) [147]. The replacement of a serine residue with a lysine residue led to killing efficiencies of around 80% for adhered *L. ivanovii* also in the early biofilm formation stage [148]. Interestingly, the temporin analog that was composed entirely of all non-natural D-amino acids decreased bacterial adhesion by 50%. The exact positioning of the grafting (N-terminus, C-terminus, or through the center) was investigated; the killing efficiency of adhered *S. epidermidis* and *E. coli* early in the biofilm formation process (3 h) was highest for the central grafting site [151]. 

Cationic peptide melimine has been extensively investigated by Kumar, et al. The impact of the peptide conformation on antimicrobial activity was investigated by connecting the peptide to a glass surface through the N-terminus, the C-terminus or in the middle as a function of positioning a cysteine residue that was reacted with a functionalized SAM (Table 4, row 3) [149]. Grafting through the N-terminus or middle position outperformed grafting through the C-terminus, with 69% and 67% reduction in coverage, respectively, versus the control for *P. aeruginosa* and 83% and 64% reduction in coverage, respectively, versus the control for *S. aureus* after 48 h. This work was carried further to perform a similar study on a titanium surface using grafting through the N-terminus of melimine [152]. When this device was implanted into a rat model, the coating reduced biofilm formation of *P. aeruginosa* and *S. aureus* by up to 62% and 84%, respectively. The authors postulate that the surface-grafted melamine operates by permeation of the bacterial membrane by the cationic portion of the peptide, although given that this peptide is effective against both Gram-negative and Gram-positive bacteria, this mechanism of action may be multifaceted.

AMP HHC36, which had been PEGylated and functionalized with an azide, was grafted to a titanium surface with a tethered alkyne through the use of the copper-mediated click reaction (Table 4, row 4) [150]. The functionalized titanium surface was exposed to a culture of *S. aureus* and inhibited 86% of bacterial growth after 48 h and 70% of bacterial growth after 96 h. This same group continued to investigate HHC36 by creating a fusion peptides with BFP-1, which has been shown to promote osseointegration, and binding those to titanium surfaces through a cysteine at the N-terminus [153]. The two peptide sequences were separated by a PEG spacer that was systematically varied in length from 0 to 24 repeat units, and the antimicrobial activity against *S. aureus*, methicillin-resistant *S. aureus*, *S. epidermidis*, *E. coli*, and *P. aeruginosa* was measured. Except for the 24-unit spacer fusion peptide, all showed strong antimicrobial activity and suppressed biofilm growth to 10–30% of the level of the control. 

The AMP GL13K, developed from a human salivary protein, was immobilized on titanium through conjugation to a SAM of (3-chloropropyl)triethoxysilane (CPTES) and tested against *Streptococcus gordonii* [154]. *S. gordonii* is a bacteria that is commonly found in the mouth and which, in turn, promotes biofilm growth by *Porphyromonas gingivalis*. The authors designed this experiment to simulate a surface that was relevant to a dental implant. The biofilm growth by *S. gordonii* after 3 days was significantly inhibited as determined by LIVE/DEAD staining and SEM imaging. Interestingly, high resolution SEM imaging of the biofilms showed that the bacterial cell walls suffered much more damage when this experiment was conducted in a bioreactor in which the bacteria underwent more division than when the assay was conducted under mild agitation in an orbital shaker.

Silicone catheters were grafted with a silanol-functionalized version of the short cationic antimicrobial peptide Palm-Arg-Arg-NH2 and evaluated against *S. aureus* for biofilm growth over a period of 30 days [155]. The peptide-functionalized silicone outperformed the unfunctionalized silicone control significantly for the first two weeks, and moderately up to one month. The AMP-functionalized catheter was also compared to a commercially available catheter with a Ag^+^-chelated surface. The peptide-functionalized silicone outperformed the commercially available catheter over the initial two-week period of the study. However, the commercially available Ag^+^-chelated catheter showed more biofilm inhibition in the last half of the 30-day evaluation period.

### 5.5. SAMs Tethering Metal Cations and Nanoparticles

In addition to grafting antibiotics and peptides to self-assembled monolayers, metal cations and nanoparticles (NPs) that are known for their antibacterial effects [156] have been grafted to SAMs on a variety of surfaces for the disruption of biofilm formation. Silver ions and nanoparticles in particular have been used extensively for antimicrobial applications in consumer products such as clothing fabrics, personal care products, and wound dressings [157,158]. Silver has a broad spectrum of antimicrobial activity, and if it is used in small amounts to minimize cost and toxicity issues, silver can be an effective microbicide. Multiple recent reviews focus on how silver has been employed and its mechanisms of action [159,160,161,162].

Silver cations were coordinated to titanium and stainless steel surfaces through a phosphonic acid SAM terminated in a thiolate anion (S^−^) (Table 5, row 1) [163,164]. The substrates were evaluated against *E. coli*, *S. aureus*, *S. epidermidis*, and *P. aeruginosa*. This silver-coordinating SAM inhibited bacterial cell adhesion by three orders of magnitude and suppressed biofilm formation by 80% compared to the control after 3 days (up to one week in the case of *E. coli*). LIVE/DEAD staining indicated that the few bacteria that did adhere to the surface were dead, suggesting that the SAM coating both kills bacteria and prevents their adhesion [164]. The amount of silver that is necessary for this effect is less than 1 nmol/cm^2^, which is lower than many other anti-microbial silver treatments. This SAM was evaluated in vivo subcutaneously in mice against *S. epidermidis* and was found to reduce adherent bacteria 500-fold after 14 days.

In addition to stainless steel and titanium surfaces, hydroxyapatite (HA), a biocompatible material that is used as a coating for implants and as a bone substitute, has been used as a surface (Table 5, row 2) [99]. Chains of 11- and 16-phosphonoundecanoic acid terminated by a negatively charged carboxylic acid were self-assembled on HA, followed by the attachment of silver. The surfaces were incubated for 3 h with *S. aureus* and evaluated by counting CFUs. Both the short and long chains reduced the CFU count by a factor of about three as compared to the control HA surface, indicating that the SAM length was insignificant to bacterial adhesion. The authors suspect this lack of difference was due to similar amounts of silver that were adsorbed to both surfaces. The impact of various sterilization methods on these surfaces was also explored. Silver-short chain SAM surfaces were most degraded by oxygen gas plasma, while silver-long chain SAM surfaces were equally degraded by oxygen gas plasma, dry heat, and UV irradiation. 

Copper also has antibacterial properties, and Cu^2+^ cations have been grafted to a monolayer of 2,2′-bipyridine that was covalently bound to glass (Table 5, row 3) [165]. To measure effectiveness against *S. aureus* and *E. coli*, each strain was deposited on modified and unmodified glass slides which were then topped with coverslips; this two-slide piece was suspended in PBS to prevent growth due to media presence and incubated for 5 h and 24 h. The microbicidal effect (ME) was determined as the log(CFU mL^−1^) of the unmodified glass slide minus the log(CFU mL^−1^) of the modified slide. An ME of 4 means 99.9% of bacteria were eliminated upon surface contact. At 24 h, the ME was 1.73 and 2.77 for *S. aureus* and *E. coli,* respectively. The authors contend this ME is remarkable, especially in light of the Cu content, approximately 16 ng cm^−2^. Additionally, within the first 24 h, 80% of the Cu content was released. Whereas the Cu^2+^ complex in this study was positively charged, a further study by the same group complexed Cu^2+^ to a dioxo-2,3,2 ligand to form a neutral complex (Table 5, row 4) [166]. This metal complex was then covalently bound to glass at a Cu concentration of 2.6 ng cm^−2^. To measure the antibacterial properties, the ME was determined at 5 h and 24 h for *S. aureus* and *E. coli*, as it was previously. At 24 h the ME was 0.4 for *S. aureus* and 1.2 for *E. coli*, much reduced from the previous study. While the mechanism of action is not known for certain, it is speculated that the neutral charge attracted fewer bacteria to the surface, as bacterial membranes are negatively charged. Surfaces in this study could be reloaded with Cu^2+^ through a simple dipping process; a single reloading step returned 76% of the Cu to the surface. 

A derivative of silver sulfadiazine (Ag-SD), silver sulfadiazine maleimide (Ag-SDM), was bound to a thiol-terminated SAM on glass (Table 5, row 5) [167]. Ag-SD is a sulfonamide antibiotic that that is regularly used to treat burn wounds and encourage healing. We include discussion of this SAM here because without the accompanying Ag^+^, SD and SDM do not convey antibacterial properties. At 24 h the SDM-thiol SAM produced ME values for *S. aureus* and *E. coli* of 0.2 and 0.1, respectively, while the Ag-SDM-thiol SAM gave ME values of 6.1 and 4.0, respectively. The amount of silver on the surface was minimal, about 43 ng cm^−2^, and over 48 h less than 12% Ag^+^ was released into solution. 

For further background regarding metal complexes in general and their antibiofilm activity, readers are referred to the following two reviews [168,169]. 

To the best of the authors’ knowledge, one of the first instances of grafting nanoparticles to the surface via SAMs to investigate antibacterial activity was in 2008 (Table 6, row 1) [170]. Ag^+^ ions and Ag NPs were grafted to a monolayer of N-(2-aminoethyl)-3-aminopropyl-trimethoxysilane (DIAMO) that was self-assembled on glass. This early work’s goal was partly to shed light on the mechanism of Ag’s antibacterial activity, and partly to create surfaces that do not shed their nanoparticles. *E. coli* and *Micrococcus luteus* were incubated in test tubes with coated and uncoated slides for 1, 5, and 24 h, and viable counts were determined to report the log(CFU mL^−1^). While this work did not investigate the DIAMO-AgNP’s effects on biofilm growth, it serves as an early example in the field of attaching NPs to surfaces via SAMs. Two years later, Ag nanoparticles were grafted to surfaces with self-assembled monolayers of (3-aminopropyl)triethoxysilane (APTES) (Table 6, row 2) and mercaptopropyltrimethoxysilane (MPTS) [171,172]. Ag NP-APTES SAMs were prepared on titanium surfaces and *S. aureus* and *E. coli* suspensions were incubated on the surfaces for 24 h [171]. Quantitative measurements of the CFUs indicated 94% *S. aureus* and >95% *E. coli* were killed; qualitative SEM observations of the surfaces confirmed the minimal presence of bacterial cells. Ag NP-MPTS surfaces were prepared on glass and used to investigate the effect of the release of Ag^+^, which after 19 days constituted only 15% of the Ag that was deposited on the surface [172]. *E. coli* and *S. aureus* were grown on modified and unmodified glass slides for 5 h and 24 h. The microbicidal effect (ME) was determined similarly to the method that was described previously for the Cu^2+^ 2,2′-bipyridine complex. For both bacterial strains, the surfaces were considered acceptable disinfectants at 24 h, though at 5 h, the surface was more effective against Gram-negative *E. coli*. When comparing this surface’s ME values to the Cu-coordinating SAM [165], Ag NP-MPTS surfaces were 2–3 times greater; however, per cm^2^, the Ag surfaces contained about 22 times more material than the Cu surfaces.

An additional method to secure citrate-stabilized Ag NPs to glass is through the use of a monolayer of a macrocyclic ligand for Cu^2+^ [175]. This surface resulted in a stronger ME at 24 h for *E. coli* and *S. aureus* compared to the Ag NP-MPTS surfaces that were described previously. The authors suspect this increase of 1–2 orders of magnitude resulted from a greater Ag^+^ release.

To minimize the release of NPs into the environment, which increases applicability to indwelling medical devices, Ag (Table 6, row 2) and CuS (Table 6, row 3) have been adhered electrostatically onto APTES-coated glass slides [173,174]. Both nanoparticles were shown to release their corresponding cations, with Ag^+^ exhibiting a steady release for approximately 20 days before decreasing [173] and CuS for one week [174]. The citrate-coated Ag NP surface reduced the cell viability of *S. epidermidis* by five orders of magnitude, as measured by determining the ME after 24 h of biofilm growth. Cell death near the Ag surface was confirmed using LIVE/DEAD staining with confocal laser scanning microscopy (CLSM) [173]. The CuS NP substrates were evaluated against *E. coli* and *S. aureus* for their ME. After 5 h, nearly 95% of the bacterial cells were killed, with greater than 99.9% killed at 24 h [174]. CuS offers the additional benefit of thermal heating through exposure to near infrared light. Human tissue and skin are transparent to wavelengths of light within the first biological window (700–950 nm) [176]. To explore this effect, *P. aeruginosa* was grown on CuS NP surfaces which were exposed to a 950 nm laser (irradiance 0.35 W cm^−2^) for 30 and 60 min [174]. Temperature increases due to an 800–1000 nm laser ranged from 6–9 °C after 40 s exposure time. Using a LIVE/DEAD stain showed 64% of bacteria were dead after 60 min irradiation, compared to 37.5% on the CuS NP surface without radiation. This added hyperthermic effect serves as a boost to killing bacteria. 

Photothermal therapy is a promising treatment tool due to its minimal invasiveness and ability to kill both Gram-positive and Gram-negative bacteria [177,178]. Similar to the CuS NP-coated surfaces, surfaces that were coated with SAMs of branched gold nanoparticles, called gold nanostars (Au NSs), have been shown to be capable of achieving bactericidal activity with near infrared light-induced heating [179]. These Au NSs were grafted to a monolayer of 3-mercaptopropyltrimethoxysilane (MPTS) on glass at a concentration of 2.0–3.0 μg cm^−2^. MRSA was incubated on surfaces for 16 h, after which the samples were irradiated by an 808 nm laser (irradiance 0.090 W cm^−2^) for 5, 10, and 30 min. In air, an irradiance of 0.080 W cm^−2^ corresponded to a Au NS surface temperature change of approximately 2 °C. Biofilms were scraped from the substrates, and the samples that were irradiated for 30 min experienced a reduction of CFU by two orders of magnitude compared to the plain glass slides. The presence of dead cells on the surface was confirmed using a LIVE/DEAD stain and CLSM. Au NSs without irradiance did not show antibacterial effects, nor did laser irradiance without Au NSs. Photothermal therapy has also been used with other shapes of particles including cubically-shaped Prussian blue NPs that were grafted to a monolayer of trimethoxysilylpropyl(polyethylenimine) and Ag triangular nanoplates that were adsorbed on glass that was functionalized by polyethylenimine [180,181].

To synergistically combine the antibacterial nature of Ag NPs and hyperthermic effects of Au NSs, a combination surface was explored. Au NSs were adsorbed to APTES-coated glass [182,183]. A ~4 nm SiO_2_ coating was applied to the Au NS layer, to which a thiol-terminated SAM was attached, followed by a monolayer of citrate-coated Ag NPs that were grafted via APTES. It was determined that the SiO_2_ layer acted similarly to a clean glass surface, chemically and physically separating the Au NSs from the Ag NPs, allowing the two layers to act independently and synergistically. The surfaces were incubated with *E. coli* and *S. aureus* for 24 h. Prior to irradiation, the combination surfaces showed a decrease in the cell density compared to the plain glass surfaces, anticipated due to the NP-release of Ag^+^ ions. The subsequent irradiation for 10 s with a 785 nm laser resulted in deformation and disruption of cell shape, as evidenced by SEM. No effect of irradiation was found on plain glass.

PDMS was modified with allyltriethoxysilane and combined with thiol-terminated SiO_2_ microspheres via a light click reaction (exposure to 365 nm light); Ag NPs then self-assembled on the surface of the microspheres through immersion in a AgNO_3_ solution [183]. The surfaces were incubated with *E. coli* and *Bacillus subtilis* for 24 h and fluorescently stained to visualize viability. For both bacteria strains, less than 15 cells were present on the modified surfaces, compared to around 300 cells on the plain PDMS surfaces.

For applicability to a wide variety of metal and polymer surfaces, recent work has silanized such surfaces with *N*^1^-(3-trimethoxysilylpropyl)diethylenetriamine (TMS) to create a SAM for the attachment of metal NPs including Ag [184]. No substrate preparation was necessary to link the TMS SAM to the surfaces that were explored. As an example of antibacterial activity, polycarbonate (PC) surfaces with varying number densities of attached Ag NPs were incubated with *P. aeruginosa*, *E. coli*, and *S. aureus* for 24 h. SEM observation and LIVE/DEAD assays showed the successful inhibition of all three bacterial strains, that were attributed by the authors to the gradual release of Ag^+^ ions. Ag NP-TMS-PC surfaces were also investigated for cytocompatibility with NIH3T3 fibroblast cells. The cell viability was greater than 90% for surfaces with lower densities of Ag NPs and exhibited no significant differences from the PC control surface. The highest Ag-density surface resulted in a cell viability of 84%.

More recent efforts have been made to fabricate Ag NPs through green methods to reduce the use of toxic chemicals and the impact of synthesis on the environment [185]. Ag NPs that were fabricated with silver grass leaves were grafted to a monolayer of APTES on glass [186]. Surfaces that were incubated with *P. aeruginosa* overnight were observed using SEM. The Ag NP-APTES surfaces exhibited a decrease in the cell surface coverage by a factor of three compared to the APTES-only control. Using flow cytometry, researchers also determined Ag NP-APTES surfaces contained 67.45% dead cells, while 38.69% cells were dead on APTES-only surfaces. 

### 5.6. Bactericidal Carbohydrates Grafted through SAMs

Carbohydrates play many roles in the biological world, including in the realm of biofilm growth. On one hand, carbohydrates make up a large proportion of the extracellular matrix that holds bacterial cells together in a biofilm. On the other hand, natural defense mechanisms against biofilms often include glycoconjugates, such as glycolipids. With this context, it is not surprising that SAMs have been employed to functionalize surfaces with carbohydrates to prevent biofilm formation.

**Figure 7:**
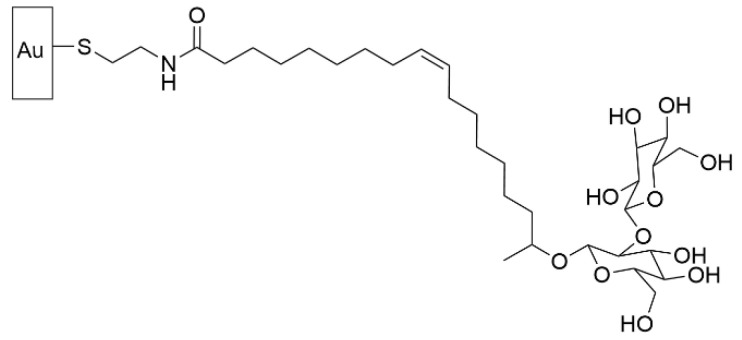
Sophorolipid-containing SAM [187].

Sophorolipids are long-chain fatty acids that terminate in saccharides and have been shown to have antimicrobial properties while possessing the advantages of low toxicity and high biodegradability. A SAM that was terminated in a sophorose (glucose *β*(1–2)) carbohydrate that was attached glycosidically to an oleic acid was prepared on an Au surface (Figure 7) [187]. This coating killed 40–50% of *L. ivanovii* upon contact, with SEM and AFM imaging showing significant damage to the bacterial cells. When the sophorose was conjugated to a saturated stearic acid chain, the SAM lost its antimicrobial activity, indicating the importance of both the carbohydrate and the unsaturation in the chain to be biocidal. The sophorolipid-terminated SAM was found to promote membrane damage after 3 h in 20–45% of the populations of Gram-positive *E. faecalis*, *S. epidermidis*, and *Streptococcus pyogenes* as well as Gram-negative *E. coli*, *P. aeruginosa*, and *Salmonella typhimurium* [188]. By further systematically varying the headgroup, it was determined that activity is carbohydrate-dependent and disaccharides outperformed monosaccharides [92]. The study hinted that only some of the hydroxyl positions were responsible for the biocidal activity, although due to limitations of obtaining the carbohydrate-functionalized lipids from biological sources, this particular aspect could not be systematically evaluated.

## 6. Outlook

Looking forward at the field, there are interesting ideas for combining different modalities of controlling bacterial growth that could afford synergistic approaches to inhibit biofilm formation. For example, a recent paper has described grafting vancomycin to a titanium surface via a peptide, GSWELQGSGSC, that is selectively cleaved by the SplB enzyme that is released by *S. aureus* [189]. This material, therefore, releases the small molecule antibiotic only when there is an active *S. aureus* infection and was shown to inhibit *S. aureus* growth more effectively than a vancomycin SAM without the cleavable linker. Although biofilm growth was not specifically investigated in this study, these results are intriguing. 

In another example, the electric potential of the surface was used to control the surface properties of a low-density mixed SAM that was prepared from a disulfide molecule in which one side terminated in an anionic carboxylate group and the other chain terminated in a cationic quaternary ammonium group [190]. When the surface is under negative potential, the cationic quaternary ammonium terminal groups “bend over” so that the cation is coordinated to the negative surface leaving only the anionic carboxylate terminal group exposed to the bacteria. As *E. coli* have positive charges along the outer bacterial membrane, *E. coli* cells are attracted to the exposed carboxylate ions and coordinate to the surface. However, when the electric potential is switched to positive, the situation reverses. The anionic carboxylate groups terminal groups become attracted to the surface, and the cationic quaternary ammonium groups are repelled by the surface and exposed to the bacteria, resulting in membrane rupture and bacteria death. Under neutral open circuit voltage, both the carboxylate and quaternary ammonium ions are exposed to the bacteria, resulting in a zwitterionic surface that repels *E. coli*. *E. coli* were exposed to the surfaces under electric potential for one hour, so at best these experiments only looked at early biofilm formation. The growth of Gram-positive *B. subtilis* was not as impacted by the electric potential switching as Gram-negative *E. coli*. Nevertheless, the idea of controlling biofilm development by switching a surface between bacterial adherent, bactericidal, and “self-cleaning” modes is exciting, especially for designing systems that can promote the detachment of established mature biofilms.

While it has not been mentioned at length in this review, many of the works that were discussed above include important experiments that were designed to evaluate the compatibility of the surface treatments with a mammalian host to determine whether the proposed coating could have a future as a viable patient treatment option. One particular work that was discussed above, has taken this idea a step further to incorporate a promoter for osteointegration into the antimicrobial peptide [91]. While the reader is referred to the text above and the article itself for more information, we wanted to call attention to this creative and synergistic approach in this section as another example of a forward-looking innovation in combatting biofilm formation.

As we have reviewed the literature that were referenced herein, one challenge in this field that has stood out is the lack of standardization for quantifying biofilm growth. This is a problem in the field of antimicrobial materials in general, and there have been recent reviews calling for more standardization [191], as well as government-issued standard operating procedures [192] and academic research articles [193,194,195] attempting to address this issue. However, many of these tests are for biocides and biofilm inhibitors that operate in vitro rather than specifically at a surface. Looking ahead, the development of more assays specifically for surface coatings will be a major step forward.

This lack of standardization in quantifying biofilm growth has clinical implications as well. Microscopic biofilms may be difficult or impossible to detect without device removal while still causing significant infection and complications for the patient, especially for infections on long-term indwelling medical devices [74]. Further research could contribute to surfaces that can prevent, and also detect, biofilm formation to provide clinicians with additional information and early detection of potential complications.

## Figures and Tables

**Table 1:** Common bacterial strains that are associated with long- and short-term IMDs.

Indwelling Medical Device	Commonly Isolated Bacteria
*Long-Term Devices*	
Orthopedic implants [43,44,45,46,47,48]	*K. pneumoniae*
	*A. baumannii*
	*S. epidermidis*
	*S. aureus*
Stents [49,50]	*E. coli*
	*Enterobacter*
	*Klebsiella*
	*P. aeruginosa*
	*E. faecalis*
	*Streptococci*
	*S. aureus*
	*S. epidermidis*
Cochlear implants [51,52]	*P. aeruginosa*
	*S. pyogenes*
	*S. epidermidis*
	*S. aureus*
Breast implants [53,54,55,56,57,58]	*E. coli*
	*Mycobacterium*
	*S. epidermidis*
	*S. aureus*
	*Streptococci*
	*Bacillus*
*Short-Term Devices*	
Urinary catheter [42,59,60]	*E. coli*
	*P. aeruginosa*
	*K. pneumoniae*
	*A. baumannii*
	*Enterobacter*
	*S. epidermidis*
	*E. faecalis*
Central venous catheter [61,62]	*P. aeruginosa*
	*K. pneumoniae*
	*S. epidermidis*
	*S. aureus*
	*E. faecalis*
Endotracheal tube [63,64,65,66]	*P. aeruginosa*
	*K. pneumoniae*
	*Acinetobacter*
	*Enterobacter*
	*S. aureus*
	*E. faecalis*
Feeding tube [67,68]	*Pseudomonas*
	*Enterococci*
	*Bacilli*
	*Staphylococci*
Contact lenses [48,59,69]	*E. coli*
	*P. aeruginosa*
	*S. aureus*

**Table 2:** Molecules that are used to form SAMs in preventative strategies for biofilm inhibition.

SAM Precursor Molecule	Surface	Reference
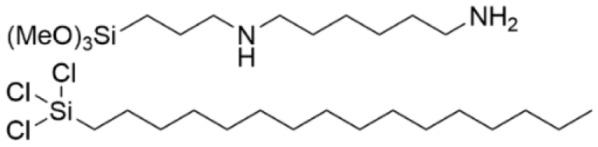	Si	[108]
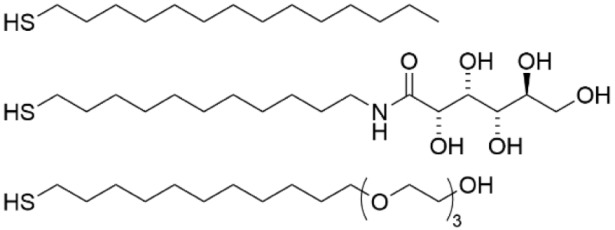	Au	[109]
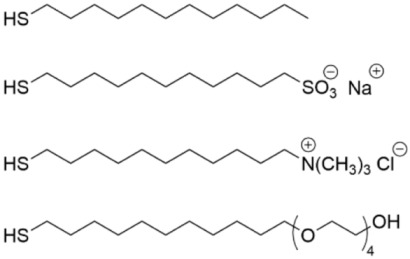	Au	[110]
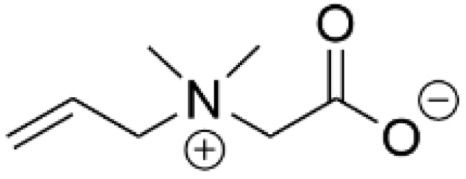	PDMS	[111]

**Table 3:** Quaternary ammonium-containing molecules that were used to form SAMs to inhibit biofilm formation.

SAM Precursor Molecule/SAM Structure	Surface	Reference
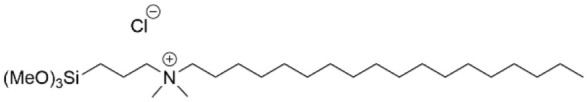	Silicone Ti	[125] [126]
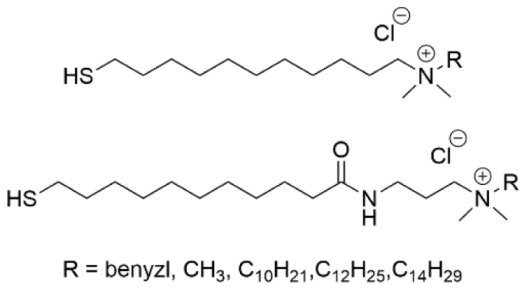	Au	[127]
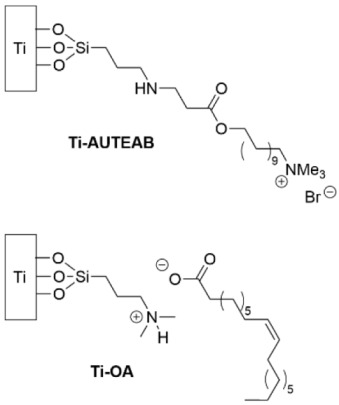	Ti	[128]

**Table 4:** Antimicrobial peptide-containing SAMs that were used to inhibit biofilm formation.

SAM Structure	Reference
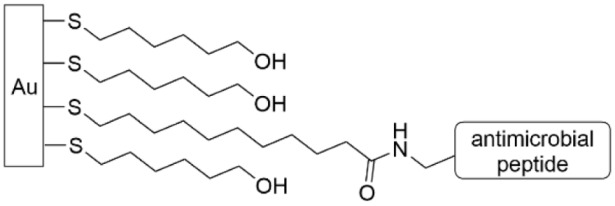	[146,147,148]
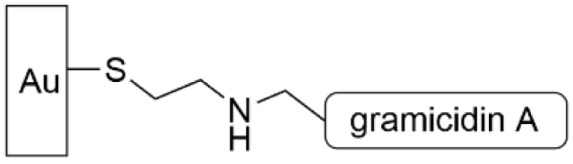	[145]
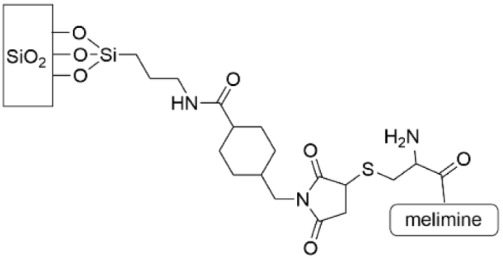	[149]
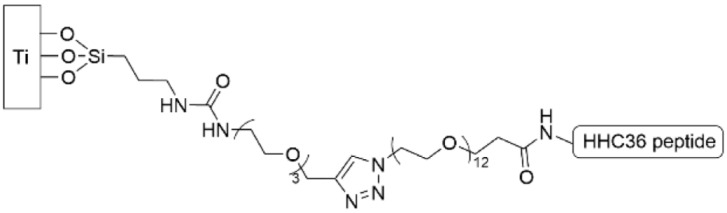	[150]

**Table 5:** Metal cations that were coordinated to SAMs to inhibit biofilm formation.

SAM Structure	Reference
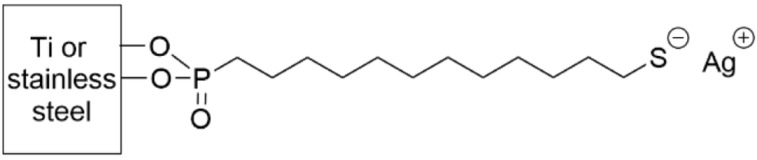	[163,164]
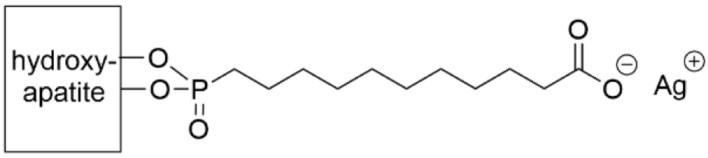	[99]
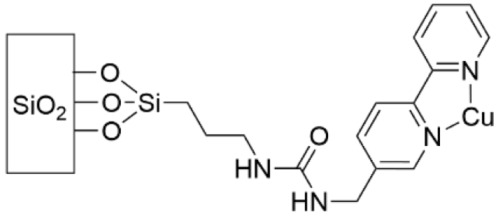	[165]
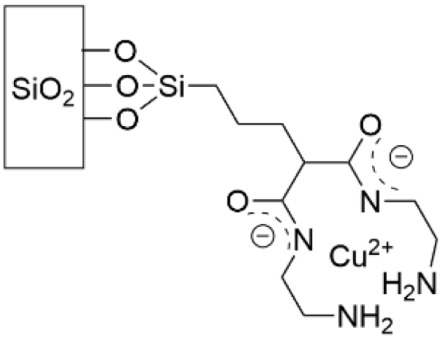	[166]
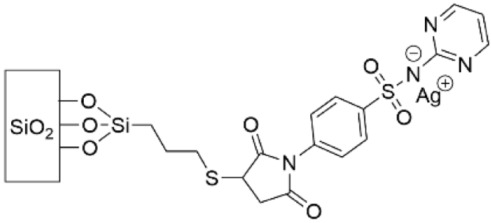	[167]

**Table 6:** Metal nanoparticles that were coordinated to SAMs to inhibit biofilm formation.

SAM Structure	Reference
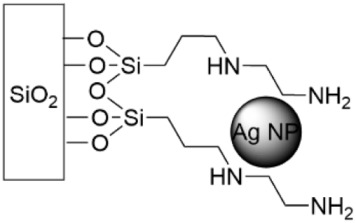	[170]
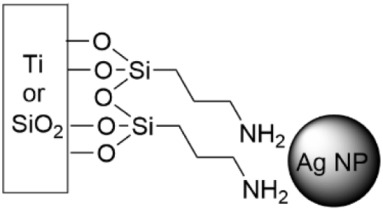	Ti: [171] Glass: [173]
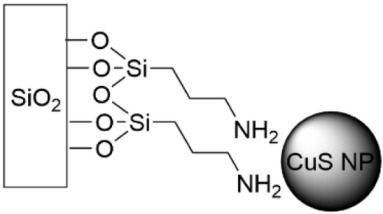	[174]
